# Mortality After Delay of Adequate Empiric Antimicrobial Treatment of Bloodstream Infection

**DOI:** 10.3390/jcm9051378

**Published:** 2020-05-07

**Authors:** Merel M. C. Lambregts, Roos Wijnakker, Alexandra T. Bernards, Leo G. Visser, Saskia le Cessie, Mark G. J. de Boer

**Affiliations:** 1 Department of Infectious Diseases, Leiden University Medical Center, 2333ZA Leiden, The Netherlands; r.wijnakker@lumc.nl (R.W.); l.g.visser@lumc.nl (L.G.V.); M.G.J.de_Boer@lumc.nl (M.G.J.d.B.); 2 Department of Medical Microbiology, Leiden University Medical Center, 2333ZA Leiden, The Netherlands; a.t.bernards@lumc.nl; 3 Department of Clinical Epidemiology and Department of Biomedical Data Sciences, Leiden University Medical Center, 2333ZA Leiden, The Netherlands; s.le_cessie@lumc.nl

**Keywords:** bloodstream infection, empiric therapy, antibiotic stewardship, blood cultures, antimicrobial resistance

## Abstract

Background: Timely empiric antimicrobial therapy is one of the cornerstones of the management of suspected bloodstream infection (BSI). However, studies about the effects of empiric therapy on mortality have reported inconsistent results. The objective of this study was to estimate the effect of delay of appropriate empiric therapy on early mortality in patients with BSI. Methods: Data for the propensity score matching (PSM) study were obtained from a cohort of patients with BSI. Inadequate empiric treatment was defined as in vitro resistance to the antimicrobial regimen administered &lt;6 h after blood cultures were taken. The primary outcome measure was 14-day mortality. Thirty-day mortality and median length of stay (LOS) were secondary outcomes. PSM was applied to control for confounding. Results: Of a total of 893 included patients with BSI, 35.7% received inadequate initial empiric treatment. In the PSM cohort (n = 334), 14-day mortality was 9.6% for inadequate antibiotic treatment, compared to. 10.2% in adequate empiric treatment (p = 0.85). No prolonged median LOS was observed in patients who initially received inadequate therapy (10.5 vs. 10.7 days, p = 0.89). Conclusions: In this study, we found no clear effect of inadequate empirical treatment on mortality in a low-risk BSI population. The importance of early empiric therapy compared to other determinants, may be limited. This may not apply for specific subpopulations, e.g., patients with sepsis.

## 1. Introduction

Bacterial bloodstream infections (BSI) have an increasing incidence worldwide and are associated with considerable morbidity and high mortality rates [1,2]. Delays in appropriate treatment of such infections may negatively affect patient outcome. To ensure adequate treatment while awaiting blood culture results, initiation of broad-spectrum antibacterial therapy is considered to be the cornerstone of the medical management of BSI [3]. In an era of ever-increasing antimicrobial resistance (AMR) rates, a recurrent discussion occurs about whether standard empiric antibiotic treatment regimens for suspected BSI should be adjusted to a broader spectrum [4,5]. Knowledge of the effects of appropriate or inadequate initial empiric therapy on patient outcome is essential to weigh the pros and cons of upscaling empiric therapy [6].

In previous studies inadequate empiric antimicrobial treatment was found to be associated with mortality. This association appeared to be stronger in critically ill patients or patients with ventilator-associated pneumoniae in combination with a BSI [7,8]. However, for obvious ethical reasons, studies on the effects of inadequate antibiotic therapy never applied a randomized, placebo-controlled design and, therefore, suffer from confounding [9,10,11,12]. A meta-analysis of prospective observational studies performed by Paul et al. in 2010 concluded that all-cause mortality was lower in patients receiving adequate empiric antimicrobial treatment. However, the included studies were heterogeneous, had a high risk of bias and the estimated effect on mortality was highly variable [11]. Various clinical variables, e.g., the severity of sepsis and comorbidity scores, have been described to impact on the choice of empiric treatment and lead to confounding by indication [10,11].

Propensity score matching (PSM) methodology has the potential to correct for these confounding differences in probabilities of receiving inadequate antibiotic therapy, thereby aiming to approach the outcome that would have been the result of a randomized study. The objective of this PSM study was to estimate the effect of a mismatch of at least the first administration of empiric antimicrobial treatment in patients with confirmed BSI on 14-day mortality rate in a large, longitudinal cohort study.

## 2. Methods

### 2.1. Study Setting and Population

Data for the propensity score matching study were obtained from a large longitudinal cohort study of patients with bacteremia [13], admitted in the Leiden University Medical Center (LUMC), a tertiary care and teaching hospital in The Netherlands. All adult patients (≥18 years) who presented during the study period (2013–2015) with an episode of mono-bacterial BSI, both hospital and community acquired, were considered eligible. Patients with contaminated blood cultures were excluded. To avoid misclassification, all blood cultures with coagulase-negative staphylococci (CoNS) were considered contaminated. For other bacteria, the classification as contamination was based on the assessment of the attending medical team at the time the blood culture result was reported.

The research center has a dedicated infectious diseases consultancy team, consisting of medical microbiologists and specialists’ infectious diseases, which is involved in all patients with BSI, performs bedside consultations, and advises on diagnostics and management. Standard empiric treatment for sepsis of unknown origin is a second-generation cephalosporin, combined with gentamicin.

### 2.2. Data Collection and Microbiology Methods

Data about demographic characteristics, medical history, clinical parameters, the source of infection and antimicrobial treatment were retrieved from the electronic patient files [14]. Clinical parameters were all collected at the time of presentation/blood culture collection and included hemodynamic parameters. The severity of illness was assessed by calculating the Pitt bacteremia score (PBS) and the quick sequential organ failure assessment score (qSOFA) score [15]. If follow up in the research center was less than 30 days, the data on survival could be traced via the electronic patient file, which is linked to the Dutch Personal Records Database (BRP).

Blood culture data, including antimicrobial susceptibility patterns, were collected from the database of the Department of Medical Microbiology. In the study center, blood cultures were analyzed using the BACTEC FX continuous monitoring system (Becton Dickinson B.V., Breda, The Netherlands). Antimicrobial susceptibility testing was performed with the VITEK2 system and E-tests (BioMérieux, Brussels, Belgium). Extended-spectrum beta-lactamase (ESBL) positivity was determined with the disc diffusion test. Minimum inhibitory concentration (MIC) breakpoints for resistance were determined according to The European Committee on Antimicrobial Susceptibility Testing (EUCAST) criteria [16].

### 2.3. Study Definitions

The primary outcome was 14-day all-cause mortality. Mortality at two weeks was chosen because the impact of inadequate antimicrobial therapy is potentially higher in the first weeks of follow-up [17]. The secondary endpoints were 30-day all-cause mortality and length of hospital stay after diagnosis of BSI. The day of the blood sampling that resulted in a positive blood culture was designated as day 0.

Initial empiric therapy was defined as the antibiotic treatment administered within 6 h after blood culture collection. This antimicrobial regimen can be regarded as an indicator for approximately the first 24 h of treatment as regimens are often optimized thereafter based on culture results or clinical course of the infection. Discrimination between adequate and inadequate initial empiric antimicrobial therapy was based on the in vitro susceptibility of the pathogen isolated in the blood culture. Adequate empiric treatment was defined as in vitro susceptibility of the isolated pathogen to at least one of the antibiotics administered within 6 h after drawing blood cultures. When no antibiotics were administered within 6 h after the blood culture collection, the initial empiric therapy was also regarded as inadequate.

Pathogen-related factors, such as virulence traits are crucial elements which may affect the clinical outcome in BSI. Based on pathogen characteristics and previous literature, pathogens were classified as low- or high-risk pathogens. Enterobacterales, *S. aureus*, *Streptococcus* spp. *ans Pseudomonas* were defined as high risk.

The BSI was considered hospital-acquired if the first positive blood culture was collected after ≥48 h of hospitalization. Prior colonization or infection with a multidrug-resistant organism was defined as the previous isolation of one of the following pathogens from any body site, including rectal swabs: vancomycin-resistant enterococci, methicillin-resistant *Staphylococcus aureus*, Enterobacterales with in vitro resistance to aminoglycosides, second- and/or third-generation cephalosporins and/or quinolones, *Pseudomonas aeruginosa* with resistance to third-generation cephalosporins, aminoglycosides or quinolones [5].

### 2.4. Statistical Methods

Categorical variables were reported as numbers with percentages and compared between the treatment groups using a chi-squared or Fisher’s exact test. The Wilcoxon rank sum test was used for comparison of respectively the distributions and medians of continuous data that were not normally distributed. Means of normally distributed continuous variables were compared using the *t*-test. Odds ratios (OR) with a 95% confidence interval (95%CI) and/or *p*-values were calculated as appropriate for each variable. The frequency of missing data was assessed, but missing data were not imputed [18].

To adjust for confounding, PSM was used to compare primary and secondary outcome parameters between patient groups that did, and those that did not, receive adequate empiric antimicrobial treatment (see below). PSM can be used to analyse observational data concerning a specific treatment outcome by identifying which individuals have the same probability of receiving the intervention (here: inadequate antibiotic treatment for the BSI). By assessing the outcome in relation to the intervention for patients with similar (i.e., matched) propensity scores, it is aimed to attain an estimate that approximates the outcome of a randomized study [19].

The propensity score is the estimated probability (0–1) of receiving inadequate antimicrobial therapy based on measured confounders. Propensity scores were generated using a multivariable logistic regression model. Variables that were included in this model were defined by univariate analysis (*p* < 0.2). The selected variables were associated with the attribution of inadequate initial empiric treatment and/or 14-day mortality. A manual backward stepwise approach was used to remove co-linear variables. The model was evaluated by using the C-statistic. A 1:1 propensity score-matching algorithm without replacement and a maximum probability distance (caliper) of 0.2 was applied. Thus, in the matched cohort a patient that did receive adequate empiric treatment was included for each patient that did not receive adequate empiric treatment, based on the propensity score. To balance baseline variables between groups of patients, calibration was performed to obtain a maximum standardized difference (SDD) of 0.10 (10%) for each covariate.

In the matched cohort, each comparison of endpoints between groups was performed by assessment of the average treatment effect in the treated population (ATT).

With the complete dataset, an analysis based on inversed probability weighting of the propensity scores (IPW) was performed as a sensitivity analysis, i.e., to assess the robustness of the results obtained by PSM. All statistical analysis were performed using STATA v.14.0 (StataCorp, College Station, TX, USA).

### 2.5. Ethical Approval

The study was approved by the Institutional Ethics Review Board of the LUMC. The results are reported according to the STrengthening the Reporting of OBservational studies in Epidemiology (STROBE) statement for observational studies and a checklist of proposed guidelines for the reporting of PSM [20]. Research data were pseudonymized and securely stored, according to the General Data Protection Regulation (GDPR).

## 3. Results

### 3.1. Cohort Characteristics

Of 897 observed episodes of BSI, four episodes were excluded because data about the empiric antimicrobial treatment were missing. Less than 2% of the variable information was missing. Of the 893 included BSI episodes, 319 (35.7%) initially received inadequate empiric treatment. The second dose usually administered after 8–12 h, remained inadequate in 89.0% of these patients in the original and in 88.6% in the matched cohort. The remaining 574 (64.3%) patients directly received adequate empiric treatment. Overall, 14-day mortality before PSM matching was 96/893 (10.7%) and 30-day mortality was 134/893 (14.9%). Baseline characteristics were not equally distributed over the patient groups that received adequate or inadequate empiric antimicrobial treatment. The source of infection, type of pathogen, site of acquisition of the infection and physical examination were all associated with (mis)match of empiric treatment (Table 1).

### 3.2. Source of Infection and Microbiology Data

The most frequent isolated pathogen was *Escherichia coli* (29.3%), *Streptococcal species* (18.2%) and *Staphylococcus aureus* (11%). The most common multidrug-resistant organisms (MDROs) observed were *E. coli* (*n* = 84, 28 ESBL positive), *Enterococci* (*n* = 25) and *Klebsiella species* (*n* = 21, 11 ESBL positive). There were no cases with Methicillin-resistant Staphylococcus aureus (MRSA) infection. The most frequent sources of BSI were intra-abdominal infection (28.9%), urinary tract infection (26.1%) and intravascular infections (12.5%).

Inadequate empiric antimicrobial treatment was more frequently observed in hospital acquired BSI (49.6%) than in community acquired BSI (29.2%), OR 1.34 (95%CI 1.02–1.74, *p* < 0.05).

### 3.3. Propensity Score Matching (PSM) Analysis

**Figure 1:**
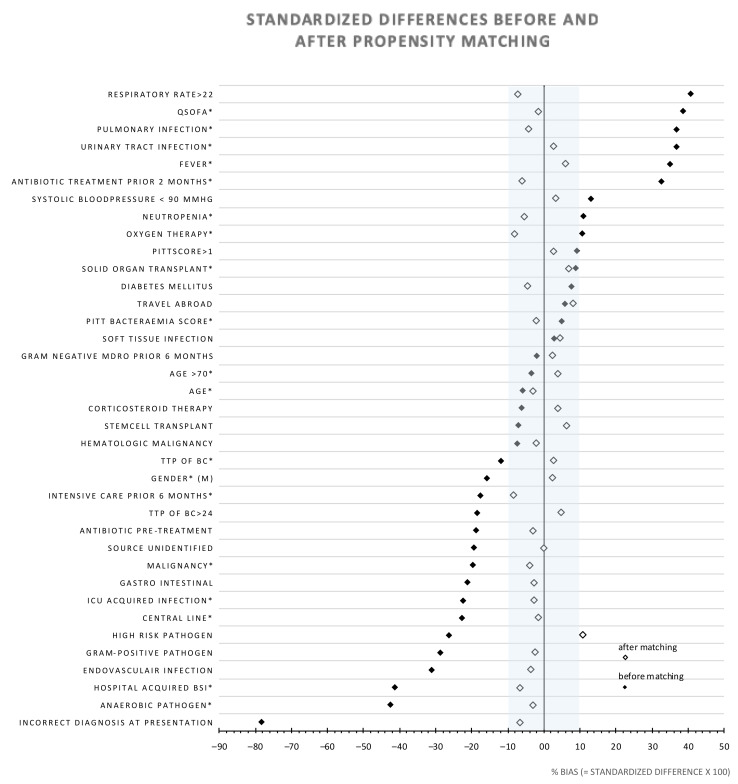
Standardized differences of study variables before- and after propensity score matching. An * indicates that the variable was included in the propensity score model. The shaded area represents the distribution with a standardized difference (SDD) <10. MDRO = Multidrug-resistant pathogen. TTP = time to positivity. ICU = intensive care unit. Fever was defined as temperature > 38.5 °C. Neutropenia: absolute neutrophil count <0.5 × 10^9^/mL.

The logistic regression model for calculation of the propensity scores consisted of 18 variables, including demographics, microbiology parameters, disease severity scores and medical history. The C-statistic of the model was 0.83. The specific variables are indicated with an * in Figure 1. After PSM, the matched cohort consisted of 334 patients, i.e., 167 matched patient pairs.

Fourteen-day mortality in the group that received inadequate empiric treatment was 16/167 (9.6%) versus 17/167 (10.2%) in the group that directly received adequate treatment (*p* = 0.85). No differences were observed in the secondary clinical outcomes among patients that initially received inadequate versus adequate treatment: 30-day mortality (21/167 vs. 25/167, *p* = 0.68) and median duration of hospital stay (10.5 vs. 10.7 days, *p* = 0.89) (Table 2).

In patients with a qSOFA ≥2, 14-day mortality was 8/41 (19.5%) in the adequate treatment group, versus 10/39 (25.6%) in the inadequate treatment group (*p* = 0.60).

After stratification for setting—hospital acquired or community acquired BSI—no effect of inadequate empiric therapy on 14-day mortality was observed, in either setting (*p* = 1.00).

The SDD for the variable ‘BSI with a high-risk pathogen’—i.e., Enterobacterales, *S. aureus*, *Streptococcus* spp. or *Pseudomonas* spp.—was 10.9%. For the remaining variables in the matched database, the SDD was <10%. The distribution of the cultured pathogens was listed per group (Supplementary Table S1). A multivariable regression analysis to adjust for this slightly unbalanced determinant showed no effect of inadequate therapy.

As a sensitivity analysis, inversed probability weighting (IPW) was performed, using the variables included in the PSM model. There was no effect of inadequate initial empiric antimicrobial treatment on mortality. The average effect of inadequate empirical treatment of 14-day and 30-day mortality was −2.2%, (95%CI −6.2–1.8, *p* = 0.29) and −3.4% (95%CI −8.0–1.3, *p* = 0.16) respectively.

## 4. Discussion

### 4.1. Key Results

In this study, empirical inadequate empiric antibiotic treatment was not associated with increased 14-day mortality in patients with BSI after applying propensity score matching methods to correct for confounding. No statistically significant differences in length of hospital stay or 30-day mortality were observed between patient groups that did and did not receive adequate empiric antimicrobial treatment. The low average Pitt bacteremia and qSOFA scores show that the majority of patients were only mild to moderately ill. Hence, the interpretation of these findings would be that these patients with BSI, an initial mismatch of the antimicrobial treatment and the susceptibility of the causing pathogen may have limited consequences. Notably, in 89% of patients with an inadequate first dose of empiric antimicrobials, the second administration was also not adequate, indicating that in most patients, the duration of time without antibiotic treatment was more than 6 h. The results of this study are in contrast to a propensity-based study by Retamar et al., in which inadequate empiric treatment was associated with increased mortality. Two methodological differences likely explain the contradicting results. Retamar et al. predominantly included patients with sepsis, including septic shock. The impact of inadequate empiric treatment is this group may be relatively high compared to the impact in patients with a lower risk for death. The low average Pitt bacteremia score and qSOFA (Table 1) in the current study shows that the majority of patients were only mild to moderately ill. Secondly, Retamar et al. choose a 4-fold longer time window, 24 h, to define inadequate empiric therapy. The prolonged time without adequate antibiotic therapy and the high proportion of sepsis/septic shock most probably are multiplicative factors driving the higher mortality associated with inadequate empiric therapy [21,22].

Other studies on the relevance of empiric antibiotic therapy also suggest that inadequate therapy leads to unfavorable outcome in BSI [6]. These studies did not apply PSM and are likely to be hampered by confounding. The complexity of the confounders that influence the adequacy of empiric treatment are illustrated in this study and stress the importance of methodology to correct for the propensity of (in)adequate treatment [11,23]. A propensity score cannot replace a randomized control trial, but such a design is unethical in this specific condition and studies using propensity scores can be considered the next best alternative in many cases.

### 4.2. Propensity of Inadequate Empiric Treatment

The adequate empiric treatment rate in this study was 64.3%. Both the adequate treatment rate and predictors for inadequate empiric therapy were comparable to previous studies investigating treatment for BSI [24]. Hospital-acquired BSI, antibiotic pre-treatment and previous hospital admissions are known risk factors for antimicrobial resistance and, therefore, risk factors for a mismatch in empiric treatment [25]. Colonization with an MDRO was not associated with a mismatch, most likely because colonization is taken into account by the attending medical team when they select empiric therapy. Low PBS scores and low SOFA scores were both associated with an increased risk of receiving inadequate empiric antimicrobial therapy [24]. This can be due to the tendency that a physicians’ tolerance of a potential mismatch of empiric antibiotic therapy is probably higher in patients who are not acutely ill and lower in patients who fulfill the criteria of sepsis.

### 4.3. Study Strengths and Limitations

This study is one of the first studies that applied PSM to assess the effect of early adequate empiric antimicrobial therapy on mortality. As illustrated in this study, whether a patient receives appropriate antibiotic therapy is subject to many variables and, therefore, (uncorrected) confounding is a major issue in previous studies. Propensity score analyses have been demonstrated to effectively reduce bias in baseline characteristics when assessing treatment effects [19]. However, in contrast to randomization, unobserved confounders may still be an issue in PSM. For example, in the present study, data on other management variables that may impact mortality, such as source control, were not available. However, measured variables, that were not included in the propensity score model, were well balanced after matching.

In The Netherlands the prevalence of MRSA is low. This may limit applicability of the results to settings in which MRSA infections are more frequent. A second limitation is that this study focuses on 14- and 30-day mortality. Inadequate antibiotic therapy may have other relevant unfavorable (long-term) effects, that were not assessed in this study [7]. Furthermore, this study does not account for suboptimal dosing of the antibiotic in the definition of adequate empiric therapy [6].

### 4.4. Generalizability and Implications

In the study cohort, the proportion of patients with sepsis or septic shock was relatively low. The results are, therefore, not applicable to selected high-risk populations. Importantly, patients present with a clinical syndrome. The exact source of infection, the yet unknown type of pathogen, the presence of sepsis/septic shock and comorbidities, may be more important determinants on the impact of inadequate antibiotics than the presence of bacteremia. Prompt adequate antibiotic treatment remains the cornerstone of the management of patients with severe clinical infections, such as sepsis [17,21,26]. In daily clinical practice, the threshold to prescribe broad spectrum antimicrobials is often low, and ‘sepsis therapy’ is frequently administered to non-septic patients suspected of BSI to avoid the risk of mismatch in empiric treatment. This study shows that the consequences of inadequate empiric therapy may currently be overestimated in a low-risk population. Therefore, in these patients, the potential beneficial effects of broad-spectrum empiric antimicrobial treatment need to be balanced with the negative effects, such as toxicity, development of AMR and *Clostridium difficile* infections [27,28,29]. Unnecessarily broad empiric antibiotics may negatively impact mortality [30]. Tolerating uncertainty in the antimicrobial spectrum, as it is already part of today’s medical practice, can benefit both the individual patient and the community (development of AMR) [31].

## 5. Conclusions

While it is widely adopted that prompt delivery of adequate antimicrobial treatment is of great importance in BSI, data to support this in patients that are mild to moderately ill, are limited.

The findings of this study clearly indicate that in this population with BSI, a limited delay in administration of adequate empiric antibiotic therapy was not associated with increased 14-day or 30-day mortality. From an antimicrobial stewardship perspective, not pursuing a 100% coverage of the expected causative agents of BSI is an acceptable uncertainty in a patient without sepsis or septic shock.

## Figures and Tables

**Table 1:** Cohort characteristics before and after propensity score (PS) matching.

	Cohort before PS Matching Empiric Antimicrobial Treatment			Cohort after PS Matching Empiric Antimicrobial Treatment:		
	**Adequate** **(*n* = 574)**	**Inadequate** **(*n* = 319)**		**Adequate** **(*n* = 167)**	**Inadequate** **(*n* = 167)**	
	***n* (%)**	***n* (%)**	***p*^#^**	***n* (%)**	***n* (%)**	***p*^#^**
**Demographics**						
Age, mean (range)	62.1 (18–98)	63.0 (18–92)	0.41	62.2 (20–91)	61.7 (18–92)	NS
Male	327 (57.0)	206 (64.6)	0.03	100 (59.9)	102 (61.1)	NS
**Microbiology parameters**						
High risk pathogen	257 (44.9)	158 (58.0)	<0.01	82 (49.1)	91 (54.5)	NS
TTP mean no. of hours (IQR)	19.0 (13–19)	21.0 (14–21)	<0.01	19.75 (13–18)	20.17 (14–21)	0.02
Gram positive pathogen	218 (38.0)	166 (52.0)	<0.001	74 (44.3)	43.1	NS
**Hospital acquired infection**	24.9%	141 (44.2)	<0.001	63 (37.7)	58 (34.7)	NS
**Source of infection**						
Urinary tract	180 (31.4)	51 (16.0)	<0.001	35 (21.0)	37 (22.2)	NS
Gastro-intestinal	436 (76.0)	212 (66.5)	0.003	113 (67.7)	115 (68.9)	NS
Pulmonary	78 (13.6)	11 (3.4)	<0.001	12 (7.2)	10 (6.0)	NS
Endovasculair	49 (8.5)	61 (19.1)	<0.001	23 (13.8)	21 (12.6)	NS
Soft tissue	46 (8.0)	23 (7.2)	0.70	13 (7.8)	15 (9.0)	NS
Unidentified	42 (7.3)	42 (13.2)	0.006	19 (11.4)	19 (11.4)	NS
Source correctly identified at presentation	426 (74.3)	120 (38.2)	<0.001	83 (49.7)	88 (52.7)	NS
**Risk factors for antimicrobial resistance**						
Antibiotic pre-treatment at presentation	152 (26.5)	111 (35.1)	0.007	61 (36.5)	58 (35.2)	NS
Antibiotic treatment in prior 2 months	246 (44.2)	188 (60.5)	<0.001	95 (56.9)	90 (53.9)	NS
Gram negative MDRO in prior 6 months	35 (6.1)	21 (6.6)	0.77	10 (6.0)	11 (6.6)	NS
Intensive care unit stay in prior 6 months	42 (7.3)	40 (12.5)	0.01	20 (12.0)	16 (9.6)	NS
**Medical history**						
Central intravenous catheter	90 (15.7)	79 (24.8)	0.001	34 (20.4)	33 (19.8)	NS
Corticosteroïd therapy	171(29.8)	104 (32.6)	0.41	52 (31.1)	55 (32.9)	NS
Diabetes mellitus	126 (22.0)	60 (18.8)	0.30	38 (22.8)	35 (21.0)	NS
Neutropenia	80 (13.9)	33 (10.3)	0.14	28 (16.8)	25 (15.0)	NS
Stem cell transplantation	41 (7.1)	29 (9.1)	0.30	15 (9.0)	18 (10.8)	NS
Solid organ transplantation	80 (13.9)	35 (11.0)	0.21	20 (12.0)	24 (14.4)	NS
Hematologic malignancy	57 (9.9)	39 (12.2)	0.31	23 (13.8)	22 (13.2)	NS
Malignancy (non-hematological)	95 (16.6)	74 (23.3)	0.016	32 (19.2)	33 (17.5)	NS
**Clinical presentation**						
Temperature >38.5 °C	380 (67.7)	157 (50.8)	<0.001	99 (59.3)	104 (62.3)	NS
Systolic bloodpressure <90 mmHg	111 (19.3)	46 (14.4)	0.07	26 (15.6)	28 (16.8)	NS
Respiratory rate >22/min	177 (30.8)	45 (14.1)	<0.001	34 (20.4)	29 (17.4)	NS
Pitt bacteremia score, mean (IQR)	1.26 (0–2)	1.17 (0–2)	<0.003	1.09 (0–1)	1.05 (0–1)	NS
qSOFA, median (IQR)	1 (0–2)	1 (0–1)	<0.001	1 (0–1)	1 (0–1)	NS

High-risk pathogen: Enterobacterales, *S. aureus*, *Streptococcus* spp. *or Pseudomonas*; TTP: time to blood culture positivity, defined as the time between collection of the blood cultures and the automated positive signal in the continuous monitoring system; Neutropenia: neutrophil count <0.5 × 10^9^/L at presentation. Corticosteroid therapy: use of corticosteroids during 6 months prior to presentation. IQR: interquartile range; MDRO: multidrug-resistant organism; *p*: *p*-value; #: chi-square test or *t*-test or Wilcoxon rank sum test; qSOFA: quick sequential organ failure assessment score.

**Table 2:** Outcomes after adequate and inadequate empiric antimicrobial therapy in patients with bloodstream infection using propensity score matching.

Outcome Variable	Adequate Empiric Regimen *n* (%)	Inadequate Empiric Regimen *n* (%)	Difference *n* (%)	OR ^#^	95%CI	*p* ^
14-day mortality	17/167 (10.18)	16/167 (9.58)	1 (0.60)	0.77	0.43–1.85	0.45
30-day mortality	25/167 (14.97)	21/167 (12.57)	4 (2.40)	0.78	0.42–1.47	0.45
Length of hospital stay in days *, median (IQR)	10.7 (4.6–18.2)	10.5 (4.3–20.3)	–	–	–	0.89

OR: odds ratio; 95%CI: 95% confidence interval; *: days counted after day of withdrawal of the positive blood culture; #: ORs were adjusted for type of pathogen (high-risk pathogen: Enterobacterales, *S. aureus*, *Streptococcus* spp. *or Pseudomonas* spp.); ^: OR and *p*-values were calculated by using logistic regression analyses. For comparison of the length of hospital stay a Wilcoxon rank sum test was applied.

## Supplementary Materials

The following are available online at https://www.mdpi.com/2077-0383/9/5/1378/s1, Table S1. Distribution of the isolated pathogens from blood cultures per group after propensity score matching.
