# Supplementary material for: Mortality After Delay of Adequate Empiric Antimicrobial Treatment of Bloodstream Infection
Source: J Clin Med. 2020 May 7;9(5):1378. doi: 10.3390/jcm9051378 (PMC7290963; doi:10.3390/jcm9051378)
Supplement: Supplementary file 1 [file jcm-09-01378-s001.pdf]

Supplementary file

Table S1. Distribution of the isolated pathogens from blood cultures per group after propensity score matching.

Species	Adequate empiric regimen *n* (%)	Inadequate empiric regimen *n* (%)
Streptococcus spp	19 (9.6)	20 (11.2)
Enterococcus spp	19 (9.6)	28 (16.8)
Staphylococcus aureus	21 (12.6)	13 (7.8)
Escherichia coli	50 (29.9)	37 (22.1)
Klebsiella spp	14 (8.4)	12 (7.2)
Pseudomonas aeruginosa	6 (3.6)	11 (6.6)
Enterobacter spp	4 (2.4)	13 (7.8)
Serratia spp	5 (3.0)	7 (4.2)
Proteus spp	5 (3.0)	1 (0.6)
Streptococcus pneumoniae	10 (6.0)	6 (3.6)
Anaerobes	7 (4.2)	6 (3.6)
Other	7 (4.2)	13 (7.8)
